# Comprehensive economic evaluation of enhanced recovery after surgery in hepatectomy

**DOI:** 10.1186/s12939-021-01583-3

**Published:** 2021-11-13

**Authors:** Yihan Dong, Yan Zhang, Chengcheng Jin

**Affiliations:** 1 School of Medicine and Health Management, Tongji Medical College, Huazhong University of Science and Technology, No.13 Hangkong Road, Qiaokou District, Wuhan, 430030 Hubei China; 2 Research Centre for Rural Health Service, Key Research Institute of Humanities & Social Sciences of Hubei Provincial Department of Education, Wuhan, 430030 China; 3 Department of Surgery, Tongji Hospital, Tongji Medical College, Huazhong University of Science and Technology, Wuhan, 430030 China

**Keywords:** Enhanced recovery after surgery, Hepatectomy, Economic evaluation, Social benefit, Multiple perspective

## Abstract

**Background:**

Enhanced recovery after surgery (ERAS) is attracting extensive attention and being widely applied to reduce postoperative stress and accelerate recovery. However, the economic benefits of ERAS are less clarified at the social level. We aimed to assess the economic impact of ERAS in hepatectomy from the perspectives of patients, hospitals and society, as well as identify the approach to create the economic benefits of ERAS.

**Methods:**

By combining the literature and national statistical data, the cost-effectiveness framework was clarified, and parameter values were determined. Cost-effectiveness analysis, cost–benefit analysis and cost-minimisation analysis were used to compare ERAS and conventional treatment from the perspectives of patients, hospitals and society. The capital flow diagram was used to analyse the change between them.

**Results:**

ERAS significantly reduced the economic burden of disease on patients ($8935.02 vs $10,470.02). The hospital received an incremental benefit in ERAS (the incremental benefit cost ratio value is 1.09), and the total social cost was reduced ($5958.67 vs $6725.80). Capital flow diagram analysis demonstrated that the average daily cost per capita in the ERAS group increased ($669.51 vs $589.98), whereas the benefits depended on the reduction of hospital stay and productivity loss.

**Conclusion:**

The mechanism by which ERAS works is to reduce the average length of stay, thereby reducing the economic burden and productivity loss on patients and promoting the hospital bed turnover rate. Therefore, ERAS should further focus on accelerating the rehabilitation process, and more economic support (such as subsidies) should be given to hospitals to carry out ERAS.

## Background

Enhanced recovery after surgery (ERAS) refers to the integrated measures centring on the entire disease cycle under the guidance of the multi-disciplinary team collaboration model, with the core goal of reducing the incidence of complications and accelerating postoperative rehabilitation [1]. Many studies have demonstrated the effectiveness and safety of ERAS, which reduces complication rates and length of hospital stay [2–4]. Therefore, ERAS has been widely used in research and practice in various clinical fields, including colorectal surgery, gastrointestinal surgery, urology, hepatobiliary surgery, orthopedics, and gynecology [5].

As an innovative clinical project, ERAS requires a large amount of equipment, capital and manpower. It should be preferentially cost-beneficial as well as socially beneficial to be favoured by financial hospital authorities. Published studies of ERAS economics indicates that ERAS has important economic value [6–9]. Domestic economic evaluation of ERAS for liver cancer, stomach cancer and colon cancer showed that the average total cost of ERAS group was lower than that of conventional group [7]. A meta-analysis of colorectal surgery found an average cost reduction of $3010 with ERAS, and a meta-analysis of pancreatic surgery showed a $7020 average cost reduction with ERAS [10]. However, most of these studies are based on a single perspective of patients, so ERAS has only been shown to reduce hospitalization costs for patients, while the economic benefits at the hospital and social level remain unclear. Moreover, incomplete cost components are involved. Costs are divided into direct costs, indirect costs and intangible costs. Since indirect costs and intangible costs are difficult to measure, they are not included in most studies.

In addition, due to the wide variety of ERAS protocols and their wide range of applications, there are some obstacles to the evaluation of economic benefits. According to expert consensus and guidelines, a series of ERAS items exists, which are unbalanced in terms of compliance and execution in practice [11–13]. Some so-called ERAS implement only a few items, which may lead to the abuse of ERAS. Studies have shown that strict implementation of standard ERAS pathways can lead to better health outcomes and economic benefits for patients [14]. As a result, ERAS protocols that do not meet standards are economically challenged, and some may even be economically ineffective. When evaluating the economic benefits of ERAS, it is also easy to make biased judgments that are higher or lower than the actual benefits, resulting in confusion in policy formulation and implementation.

Therefore, this study aims to assess the economic benefits of ERAS from the perspectives of patients, hospitals and society, as well as identify the approach to create economic and social benefits. Given the complexity of assessing the economic benefits of ERAS as a whole with different surgical procedures, hepatectomy was selected for our study. Hepatectomy (the ICD − 10 disease code is D18.013) is a widely used operation with heavy burden, which causes long-term stress reactions, high incidence of complications and slow postoperative recovery. It is also considered one of the most effective disease scenarios for ERAS [15, 16]. Thus, we took hepatectomy as an example for our study.

## Methods

### Study design

This study intended to conduct an economic analysis of ERAS. The economic framework and cost composition of patients, hospitals and society were identified by literature review. This study included patients undergoing hepatectomy, who were divided into two groups, one group receiving ERAS care (ERAS group) and the other group receiving conventional surgery (control group). The costs and benefits of the two groups under different perspectives will be compared to obtain the economic value of ERAS. To avoid possible risks to the extrapolation rationality of the results due to the differences in social system and price, cost parameters in China were adopted in this study.

### Literature search

**Fig. 1:**
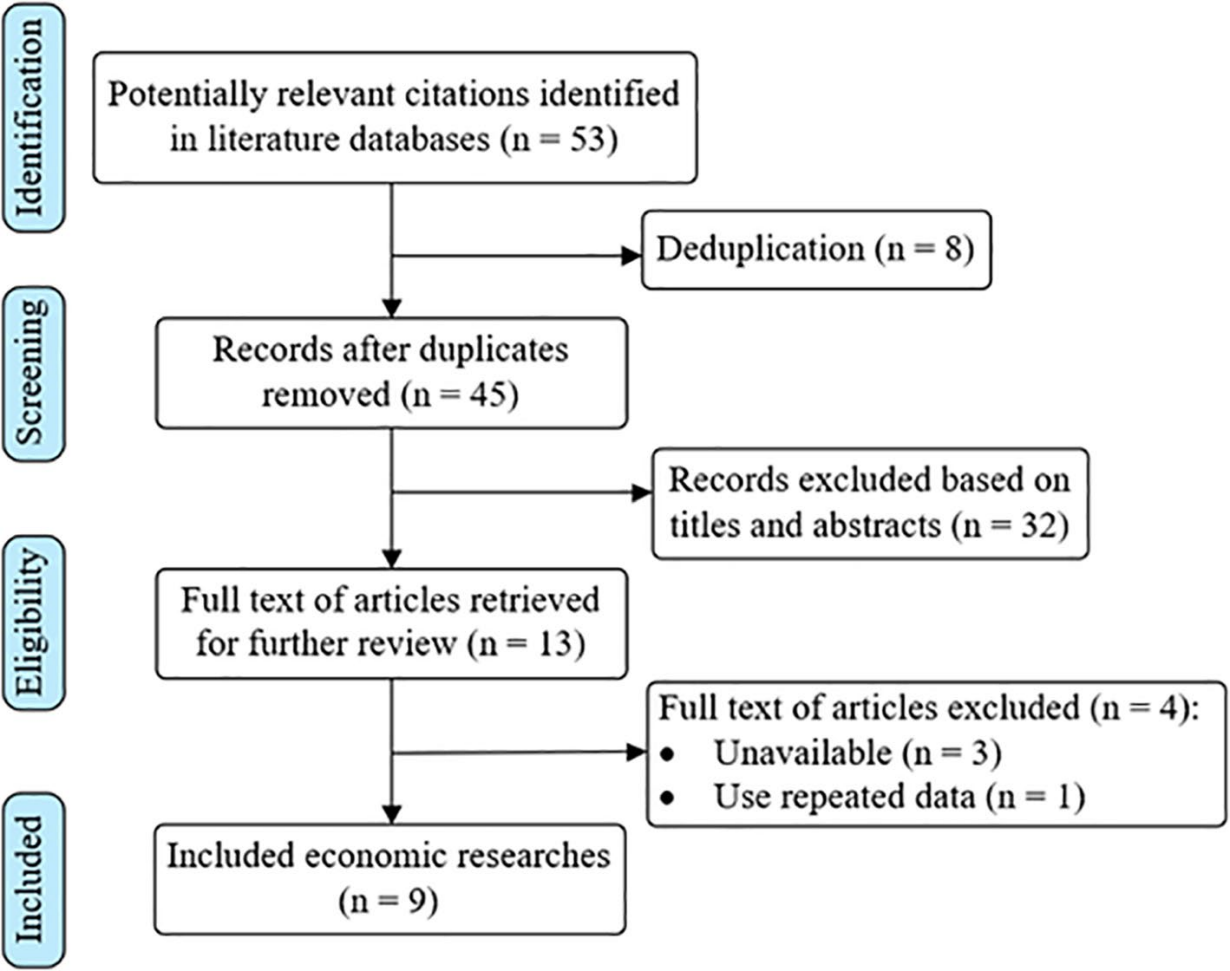
Flow diagram of study selection

A literature review was performed to construct the economic framework for ERAS. Searches were conducted in several databases, such as PubMed, EMBASE and Web of Science. Search terms included ‘enhanced recovery after surgery’, ‘fast-track surgery’, ‘ERAS’, ‘FTS’, ‘economic evaluation’ and ‘cost analysis’. Inclusion criteria: Economic evaluation of ERAS. Exclusion criteria: non-economic research, literature not available and using repeated data. After the retrieved literatures were de-duplicated, two researchers independently screened the titles and abstracts according to the literature inclusion and exclusion criteria, and then re-screened the full text. Then, the basic information of the included literature was extracted according to the pre-established data extraction form, including perspective, population, methods, indicators and results. Differences between the two researchers shall be resolved through discussion or consultation with a third party. The flow diagram of study selection is shown in Fig. 1.

### Model building and parameter determination

Cost components and effect (benefit) items and the economic conceptual model were constructed in accordance with the cost framework proposed by Larg A et al [10, 17]. The components of cost from multiple perspectives are summarised in detail in Table 1.

**Table 1:** Components of cost categorized by the type of cost from multiple perspectives

Perspective	Direct cost	Indirect cost	Intangible cost
Patients	**Direct medical cost:** hospitalisation cost (medical expenses during pre-, intra- and postoperative) **Direct non-medical cost:** food expenses; transportation; employment of care workers; others (accommodation fees, etc)	Productivity loss of patients and their families due to hospitalisation	Pain and anxiety of patient and their families caused by disease; patient medical experience and satisfaction
Hospitals	**Standard input:** routine medical services; resource consumption; administrative management **ERAS specific input:** salary of ERAS full-time nurse; quarterly ERAS meeting cost; ERAS database cost; ERAS patient log cost	–	Labor and time cost of medical staff
Society	**Hospital standard input:** routine medical services; resource consumption; administrative management **Hospital ERAS specific input:** salary of ERAS full-time nurse; quarterly ERAS meeting cost; ERAS database cost; ERAS patient log cost **Direct non-medical cost**: food expenses; transportation; employment of care workers; others (accommodation fees, etc)	Productivity loss of patients and their families due to hospitalisation	Psychosocial influence

This study estimated direct medical cost, direct non-medical cost and productivity loss. Given that the method for measuring intangible cost was of low precision, it was excluded in the cost accounting scope. As for indirect cost, since the presence or specific number of family members as caregivers could not be determined from the literature, only the productivity loss due to the patient himself/herself was considered. The incidence of complications was selected as the effect indicator, and the hospital benefit integrated medical expenses charged and bed turnover rate.

The specific parameter value was determined based on the literature and national statistical data. Considering the regional differences in consumer price index (CPI) and the impact of time on currency, the currency values were converted to the 2018 national present value on the basis of the regional and national CPI levels and discount rates for the current year [18–20]. All costs were primarily in Chinese Yuan Renminbi (CNY). The used exchange rate to US dollar (USD) was the one current on June 29, 2020: 1 CNY = 0.1413 USD.

### Economic analysis methods and statistical analysis

Cost-effectiveness analysis (CEA) was used from the patient perspective. CEA is to find a plan with the lowest cost and the best effect. The present analysis compared the cost and effectiveness of the ERAS and control groups.

Cost–benefit analysis (CBA) and univariate sensitivity analysis were conducted from the hospital perspective. CBA compares expected benefits and expected costs, using the incremental benefit cost ratio (IBCR) as the analysis index. IBCR represents the ratio of incremental benefit to incremental cost in ERAS and control groups. Univariate sensitivity analysis evaluates the influence of the changes of several major variables within a certain range on the results. Three variables were selected in this study: bed turnover rate, hospitalisation cost and hospital profit margin.

$$\mathrm{IBCR}=\left(\mathrm{B}1\hbox{-} \mathrm{B}2\right)/\left(\mathrm{C}1\hbox{-} \mathrm{C}2\right)=\varDelta \mathrm{B}/\varDelta \mathrm{C}$$

Cost-minimisation analysis (CMA) was conducted to evaluate the social resource consumption of ERAS. CMA calculated the total cost by adding all costs in ERAS group/control group and compared them.

Statistical analysis and comprehensive evaluation were performed using Excel 2016. The capital flow diagram was used to analyse changes in per capita economic burden, hospital income and total social cost. Per capita economic burden refers to the economic loss caused by disease, including direct and indirect economic loss. Hospital income refers to net revenue, which is equal to medical fees charged to patients minus hospital operating costs. Total social cost refers to the total consumption of social resources.

## Results

### Summary of parameter values

The specific parameter values are summarised in detail in Table 2.

**Table 2:** Specific parameter values ($)

Perspective	Content		Base value		Source
			Control group	ERAS group	
**Patient**	**Cost**	**Direct medical cost**			
		Hospitalisation cost	9360.56 (4854.36, 16,483.53)	7969.57 (5310.19, 10,754.38)	Jing X [7] (2018)
		**Direct non-medical cost**			
		Food expenses	824.12 (543.00, 1105.24)	742.68 (385.68, 1099.67)	Wang D [6] (2019)
		Transportation			
		Employment of care workers			
		Others (accommodation, etc)			
		**Indirect cost**^**a**^			
		Average hospital stays (d)	11.4 (8.2, 14.6)	8.9 (6.1, 11.7)	Chen L, et al [21] (2019)
		GDP per capita	9134.20/365 = 25.03	9134.20/365 = 25.03	China Statistical Yearbook [22] (2019)
	**Effect**	**Safety**			
		Complication rate (%)	15/121 × 100 = 12.4	5/79 × 100 = 6.33	Jing X [7] (2018)
**Hospital**	**Cost**	**Standard input cost**^**b**^	9360.56 × 60% = 5616.34 (2912.62, 9890.12)	7969.57 × 60% = 4781.74 (3186.11, 6452.63)	Jing X [7] (2018)
		**Specific input cost**^**c**^			
		Salary of ERAS full-time nurse	–	163.84	Joliat GR, et al [10] (2016)
		Quarterly ERAS meeting cost	–	1.34	
		ERAS database cost	–	44.52	
		ERAS patient log cost	–	1.78	
	**Benefit**	**Hospital charges**	9360.56 (4854.36, 16,483.53)	7969.57 (5310.19, 10,754.38)	Jing X [7] (2018)
		**Number of patients admitted**^**d**^	20 × 30÷11.4	20 × 30÷8.9	Chen L, et al [21] (2019)
**Society**	**Cost**	**Standard input cost**^**b**^	9360.56 × 60% = 5616.34 (2912.62, 9890.12)	7969.57 × 60% = 4781.74 (3186.11, 6452.63)	Jing X [7] (2018)
		**Specific input cost**^**c**^			
		Salary of ERAS full-time nurse	–	163.84	Joliat GR, et al [10] (2016)
		Quarterly ERAS meeting cost	–	1.34	
		ERAS database cost	–	44.52	
		ERAS patient log cost	–	1.78	
		**Direct non-medical cost**	824.12 (543.00, 1105.24)	742.68 (385.68, 1099.67)	Wang D [6] (2019)
		**Indirect cost**^**a**^			
		Average hospital stays (d)	11.4 (8.2, 14.6)	8.9 (6.1, 11.7)	Chen L, et al [21] (2019)
		GDP per capita	9134.20/365 = 25.03	9134.20/365 = 25.03	China Statistical Yearbook [22] (2019)

^a^ Patient’s indirect cost was calculated by multiplying the average length of stay by 2018 GDP per capita and dividing by 365

^b^ Hospital’s standard input (net cost) was approved by 60% of hospital charges [23]

^c^ The specific input of ERAS was converted into the purchasing power parity of 100 yuan = 40.47 Swiss francs [24]

^d^ The absolute turnover of beds was assumed in this study because of the shortage of beds in tertiary hospitals (the average utilisation rate of beds in tertiary hospitals reached 97.5% in 2018 [18])

Assuming that there are 30 days per month and the department has 20 beds [18]. The formula for calculating hospital benefit was as follows: hospital benefit = hospital charges per capita × number of patients admitted within one month = average hospitalisation cost × (20 × 30 ÷ average hospital stays).

### Results of cost-effectiveness analysis

For patients, cost-effectiveness analysis showed that the cost of ERAS group ($8935.02) was lower than that of the control group ($10,470.02), and the incidence of complications in ERAS group (6.33%) was also lower than that of the control group (12.40%) (Table 3). This suggests that ERAS has safety and economic advantages over conventional treatments.

**Table 3:** Cost-effectiveness analysis results

Group	Cost ($)	Effect (%)
**Control group**	10,470.02	12.40
**ERAS group**	8935.02	6.33

### Results of cost–benefit analysis and univariate sensitivity analysis

For hospitals, the cost and benefit of ERAS were higher than those of control group (Table 4). When the IBCR value is greater than or equal to 1, ERAS can produce positive benefits. The IBCR value in this study is 1.09, suggesting that ERAS is more cost-effective compared with conventional treatments.

**Table 4:** Cost-benefit analysis results

Group	Cost ($)	Benefit ($)	Incremental cost ($)	Incremental benefit ($)	IBCR (ΔB / ΔC)
**Control group**	295,596.84	492,661.05	41,024.73	44,613.33	1.09
**ERAS group**	336,621.57	537,274.38			

**Fig. 2:**
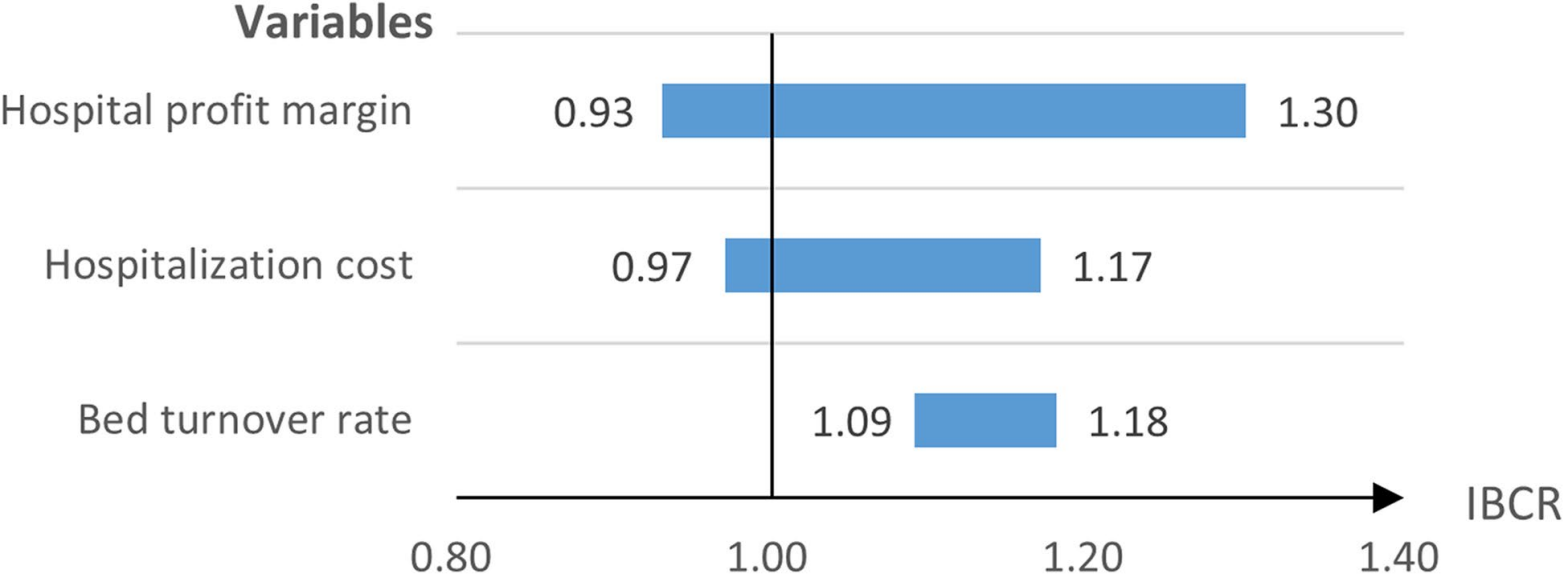
Results of univariate sensitivity analysis (tornado analysis)

Univariate sensitivity analyses were performed on three variables: bed turnover rate, hospitalisation cost and hospital profit margin. As the values of the three variables change, IBCR is still greater than 1 in most cases (Fig. 2). The univariate sensitivity analysis results indicated that the economics of ERAS protocols were relatively credible.

### Re﻿sults of cost–minimisation analysis

From the social perspective, the total cost per capita in ERAS group was $5958.67, lower than $6725.80 in the control group (Table 5). According to the principle of cost minimisation, ERAS is more economical and socially beneficial than conventional treatments.

**Table 5:** Cost-minimisation analysis results

Group	Overall cost ($)	Average hospital stays (d)	Average daily cost ($)
**Control group**	6725.80	11.4	589.98
**ERAS group**	5958.67	8.9	669.51

**Fig. 3:**
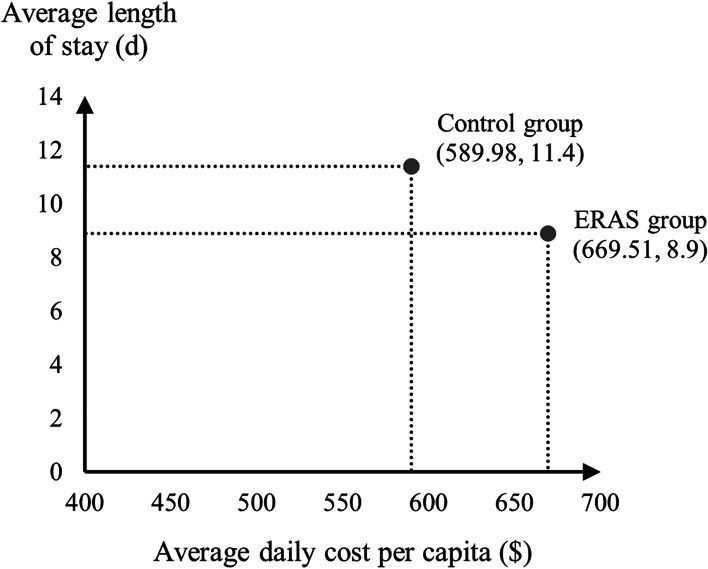
Analysis of changes in total social costs of ERAS group and control group

The average daily cost per capita in the ERAS group was higher than that in the control group ($669.51 vs $589.98). However, the total cost per capita (i.e. the area in Fig. 3) was lower in the ERAS group than in the control group due to a significant reduction in the average length of hospital stay ($5958.67 vs $6725.80).

### Comprehensive analysis of cost changes

**Fig. 4:**
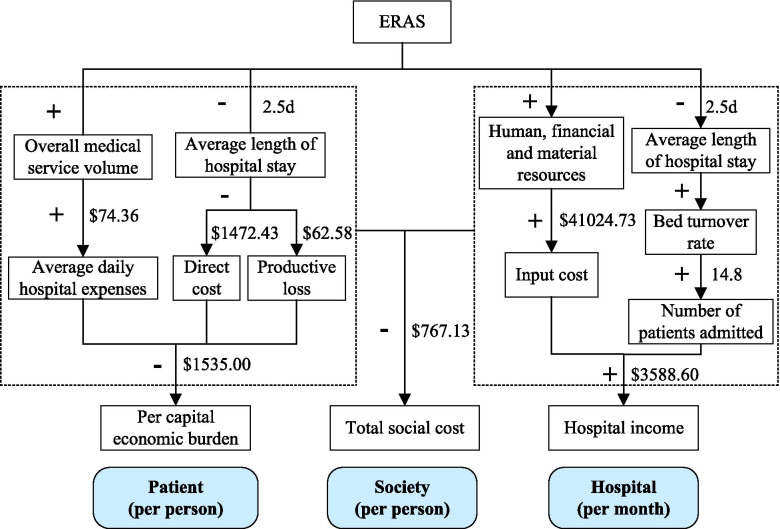
The capital flow diagram for cost variation analysis

The capital flow diagram for cost variation analysis are illustrated in Fig. 4. ERAS increases daily hospital expenses for patients, but reduces overall direct costs and productivity loss, resulting in a lower economic burden (−$1535.00). At the hospital level, there are two lines: on the one hand, the operating cost increases; on the other hand, the total medical income increases, and the final net income increases (+$3588.60). Thus, ERAS’ total cost to society is decreased (−$767.13).

## Discussion

### ERAS reduces the economic burden on patients

Our study found that ERAS significantly decreased the economic burden of patients. Patients experienced a $1535.00 cost reduction after ERAS implementation. Previous studies have shown that ERAS effectively saved $1367.51 for each person [25]. Nelson et al.’s economic evaluation of colorectal surgery found that the net cost savings attributable to ERAS ranged from $2806.00 to $5898.00 [26]. Despite the cost savings of ERAS protocols vary in different application scenarios, they all prove that ERAS is a cost-effective intervention. The findings also show that the notable contribution to cost reduction was in direct medical costs. However, the study did not report the detailed composition of medical costs. Previous studies have noted that ERAS causes significant reductions in the cost of medication and disposable consumables [27, 28].

### ERAS brings economic benefits to hospitals

In this study, the hospital input costs increased by $41,024.73 and benefits increased by $44,613.33, leading to a profit increase of $3588.60 for the hospital compared with the pre-ERAS. The economic assessment of Alberta’s ERAS plan by Nguyen et al. reported that ERAS can bring in a return of $3.8 for every $1 invested [29]. The rate of return on investment of our study (1:1.09) was lower than that of the above study (1:3.8) [29], which may be related to the late start of ERAS and the fact that medical institutions have not yet reached the optimal state of coordination and unity in various aspects. ERAS implementation costs mainly include the cost of multidisciplinary team management and training and the cost of full-time nurses [30]. However, the decrease in the input of drugs and consumables is much higher than the salary and management expenditure. As a New Zealand study reported, the cost savings due to reduced postoperative resource utilization more than offset the input costs of ERAS [31]. This means that the value of technical labor is not well reflected under the current hospital fee compensation mechanism [6, 32].

### ERAS obtains social economic benefits

Our results show a $767.13 reduction in social cost per patient receiving ERAS. A study by Lee et al. pointed out that patients managed by ERAS had less productivity loss and lower readmission rates, which contributed to the reduction in overall social cost [33]. Readmission and follow-up costs after discharge reflect the long-term economic benefits of ERAS. Richardson et al. included readmission costs in their study and found that total costs in the ERAS group were still lower than in the conventional group [34].

### Mechanism by which ERAS works is to reduce average hospital stays and increase bed turnover rate

Through the analysis of the cost variation, the economic benefits of ERAS are frequently attributed to the average length of hospital stay. The difference in average length of stay before and after ERAS translates into the cost difference, rather than the absolute length of hospital stay. The shortening of the average length of stay essentially reduces the number of ineffective hospitalisation days that are of no value to the patient and of little benefit to the hospital [35, 36]. Consequently, the consumption of social resources and the economic payment for patients are reduced. The essence of cost variation lies in the reduction of patients’ productivity loss and the consumption of ineffective services, but it increases the average daily hospitalisation cost.

Morever, the implementation of economic benefits of ERAS depends on the increase in the hospital bed turnover rate. Studies suggested that increased productivity in the health delivery system is expected to produce shorter hospital stays and lower costs [34]. Our research fully confirmed this finding. From the perspective of hospitals, the reduction in average length of stay increases bed turnover, especially in large tertiary hospitals, which are able to treat additional patients and generate more revenue with limited bed resources [37, 38]. From the perspective of society, the cost is actually transferred between patients and hospitals, and the ERAS input cost is offset by the benefits brought by the increase in bed turnover, ultimately achieving the overall reduction in consumption.

### Limitations

There are three limitations to be acknowledged. Firstly, the establishment of cost structure has a decisive impact on the result of economic evaluation, and the difference in cost framework may lead to differences in results. Secondly, a cost difference may be driven by changes in causes and conditions of hepatectomy. Here, we ignored the interference of disease severity and surgical methods on the outcome of the disease and economic impact. Finally, this study only focused on the short-term economic value of ERAS.

### Implications

ERAS offers a way to improve the efficiency of health resource use, so promoting overall patient recovery and reducing the average length of hospital stay should be a management priority in the future. Economic support is also supposed to be developed to hospitals.

Our study confirmed the economic benefits of ERAS using hepatectomy as an example, and provided an evaluation framework for the economic evaluation of ERAS. ERAS is also available in other scenarios, such as gastroenterology, gynecology, orthopedic, etc. ERAS may differ cost-effectively in different other scenarios, which will be further investigated in future studies. Similarly, to study the economic value of ERAS in other countries or regions, variations in parameters need to be considered to better match the actual situation.

## Conclusion

In conclusion, the implementation of ERAS in hepatectomy reduces the direct and indirect economic burden on patients and society, increases hospital input costs and promotes the efficiency of bed turnover. Shortening the average length of stay and improving bed turnover are the core issues in the mechanism to realise the economic benefits of ERAS.

## Data Availability

Publicly available datasets were analysed in this study. This data can be found through the search strategy.

## Abbreviations

ERAS: Enhanced recovery after surgery
IBCR: Incremental benefit–cost ratio
FTS: Fast-track surgery
CPI: Consumer price index
CNY: Chinese yuan renminbi
USD: US dollar
CEA: Cost-effectiveness analysis
CBA: Cost–benefit analysis
CMA: Cost-minimisation analysis
